# Uncovering key biomarkers, potential therapeutic targets and development of deep learning model in heart failure

**DOI:** 10.1371/journal.pone.0330780

**Published:** 2025-09-03

**Authors:** Ming Du, Shuang He, Jiaojiao Liu, Long Yuan

**Affiliations:** Department of Cardiovascular Medicine, Liaoning Provincial People’s Hospital, Shenyang, China; Sichuan University, CHINA

## Abstract

Heart failure (HF) represents a significant public health concern, characterized by elevated rates of mortality and morbidity. Recent advancements in gene sequencing technologies have led to the identification of numerous genes associated with heart failure. By utilizing available gene expression data from the Gene Expression Omnibus (GEO) database, we conducted a screening for differentially expressed genes (DEGs) related to heart failure. Key genes were identified through intersection analysis in conjunction with weighted gene co-expression network analysis (WGCNA). Following this, we pinpointed four essential genes (ITIH5, ISLR, ASPN, and FNDC1) by employing functional enrichment analyses, machine learning approaches, protein-protein interaction (PPI) assessments, gene set enrichment analysis (GSEA), and immune infiltration evaluations. Additionally, a novel diagnostic model for heart failure was successfully developed using a deep learning convolutional neural network (CNN), and its diagnostic performance was validated within public datasets. Analysis via single-cell RNA sequencing further indicated stable up-regulation patterns of these genes across various cardiomyocyte types in HF patients. Moreover, the exploration of drug-protein interactions revealed two potential therapeutic drugs targeting the identified key genes, with molecular docking offering a feasible pathway for this connection. In conclusion, we identified four potential key biomarkers closely related to HF and two possibly effective small molecules, which provide significant insights into the molecular mechanisms underlying heart failure and the search for new therapeutic targets.

## 1 Introduction

Despite significant advances in understanding the risk factors associated with incident heart failure (HF), research indicates that HF continues to represent a substantial clinical and public health challenge [1]. While lifestyle modifications, secondary prevention strategies for coronary heart disease, and effective blood pressure control contribute to HF management, there are currently no more effective primary prevention interventions for HF [2,3]. This emerging body of knowledge facilitates the identification of novel biological mechanisms linked to HF events and holds the potential to guide the development of innovative interventions aimed at the primary prevention of HF. Commonly employed diagnostic techniques for HF in clinical practice exhibit several limitations. Levels of brain natriuretic peptide (BNP) and N-terminal proB-type natriuretic peptide (NT-proBNP) may be elevated in various non-HF conditions, including infections, acute or chronic renal failure, and inflammation, while remaining normal in patients with heart failure with preserved ejection fraction (HFpEF) [4,5]. Echocardiography, a widely used technique for cardiac function assessment, primarily depends on the operational proficiency and diagnostic expertise of clinicians [6]. Additionally, it is challenging to accurately identify HFpEF patients based solely on ejection fraction (EF) measurements [7].

With the advent of next-generation sequencing, large-scale genomic and transcriptomic analyses have facilitated the discovery of novel biomarkers and underlying molecular mechanisms of HF [8–10]. Several HF-associated genes have been identified, including FHOD3, a gene linked to cardiomyopathy and HF [11]; Titin (TTN) and MYH7, which are associated with cardiomyopathy progression [12,13]; NPPA, which encodes atrial natriuretic peptide regulating fluid balance [13]; and BMP4, involved in cardiac development and signaling pathways [14]. Understanding these molecular factors offers a promising avenue for developing innovative diagnostic and therapeutic strategies.

In this research, a large amount of gene expression data obtained from the Gene Expression Omnibus (GEO) was employed to explore the fundamental biological mechanisms involved in HF. Utilizing Weighted Gene Co-expression Network Analysis (WGCNA), we pinpointed crucial module genes from the most extensive HF dataset available. This was followed by feature enrichment analysis, which facilitated the selection of four machine learning techniques to identify significant genes linked to HF. Additionally, analysis of the protein-protein interaction (PPI) network provided further insights. We developed a nomogram based on these key genes as a predictive framework, which exhibited excellent performance and was validated against an independent dataset. A deep learning model was developed, utilizing genes associated with heart conditions, ultimately aiding in clinical diagnosis.

**Fig 1:**
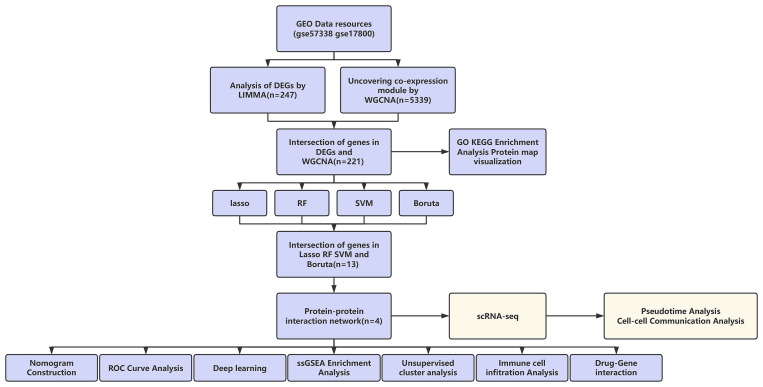
Graphical abstract of the analysis process.

Additionally, we assessed the possible relationships between immune cell populations and HF. Recent studies increasingly indicate that immune dysregulation and the decline of cardiac function are mutually reinforcing phenomena [15]. Moreover, we carried out single-cell RNA-sequencing analysis on GSE145154, thus demonstrating the expression distributions and dynamic changes of key genes in damaged cardiomyocytes of HF patients. Furthermore, molecular docking analysis was carried out to predict potential therapeutic agents targeting these hub genes. Finally, a schematic diagram illustrating the analytical workflow is presented (Fig 1).

## 2 Materials and methods

### 2.1 Data acquisition and processing

The dataset related to the disease was obtained from the GEO database [16]. For HF, we chose GSE17800 [17] and GSE57338 [18] as the main datasets. We selected GSE57338 as our primary dataset for HF analysis due to its extensive sample size, including 177 HF patients and 136 individuals without HF. The sequencing platform of GSE57338 was GPL11532. The GSE17800 includes 40 HF patients and 8 individuals without HF. The sequencing platform of GSE17800 was GPL570. The ComBat algorithm is an empirical Bayes-based method crucial in bioinformatics for correcting batch effects in high-throughput data, particularly gene expression studies [19]. The combat algorithm was applied to remove the batch effect on both samples. Whereas GSE29819 [20] served as the validation test group, including 26 HF patients and 12 individuals without HF. The sequencing platform of GSE29819 was GPL570. R software (version 4.2.0) was employed for data preprocessing. Additionally, GSE145154 [21] was employed for further validation as well as for scRNA-seq analysis, including a total of 18,730 cells from HF and a total of 15,004 cells from control.

### 2.2 Principal component analysis and identification of differentially expressed genes

To evaluate the variation between the control and HF groups, principal component analysis (PCA) was conducted. Following this, the R package “limma” (3.58.1) [22] was employed to determine differentially expressed genes (DEGs) in relation to the control group and HF samples. The cutoff standard for DEGs was |logFold Change (logFC) | > 0.585, and adjust *p* < 0.05. Volcano plots and heatmaps for the DEGs were generated using the “pheatmap” package (1.0.12).

### 2.3 Weighted gene Co-expression network analysis

To investigate gene interactions, we applied the “WGCNA” R package (1.72–5) [23] on the final integrated dataset. Initially, we selected the top 50% of genes that displayed the greatest inter-sample variation from the comprehensive WGCNA dataset, thus ensuring both heterogeneity and statistical precision for the subsequent co-expression network analysis. Next, genes with a height value exceeding 60 were classified as outliers and removed from the analysis. Subsequently, the soft threshold power β for the computed adjacency matrix was established and then transformed into a topological overlap matrix (TOM). Following this, genes were organized into specific modules via hierarchical clustering and dynamic tree cutting, utilizing TOM-based dissimilarity measures. Finally, the variability of the characteristic genes within each module was computed, and the cut-off values for the dendrogram were chosen to enable further analysis of the various modules. The genes identified through the intersection of volcano plots, heatmaps, and WGCNA methods were deemed significant for HF.

### 2.4 Functional enrichment analysis

In order to elucidate the biological processes and functions of genes related to HF, our study used the “clusterProfiler” R package (4.10.1) [24]. This tool allowed us to perform comprehensive gene ontology (GO) [25] and Kyoto Encyclopedia of Genes and Genomes (KEGG) [26] enrichment analyses. Through this analysis, we were able to identify and visualize key pathways and gene functions, providing deeper insights into how genes contribute to the pathophysiology of HF.

### 2.5 Machine learning

Four machine learning methods were employed, including Least Absolute Shrinkage and Selection Operator (LASSO) [27], Random Forest (RF) [28], Boruta [29], and Support Vector Machine Recursive Feature Elimination (SVM-RFE) [30], to thoroughly screen for potential genes linked to HF. The LASSO method was implemented through the “glmnet” package (4.1–8), utilizing ten-fold cross-validation to pinpoint significant genes. We conducted the RF analysis via the “randomForest” package (4.7–1.1), identifying the top 30 genes as potential candidates. For the SVM-RFE approach, the “e1071” package (1.7–14) was employed to ascertain the optimal gene count based on the accuracy. Meantime, the candidate genes were further validated using multiple machine learning algorithms, employing the “Boruta” R package (8.0.0). Finally, by intersecting the findings from these four different machine learning techniques, we extracted the potential genes related to HF.

### 2.6 Protein–protein interaction network construction

To gain a deeper insight into the development of HF and to explore the relationships between protein-coding genes, PPI network was established employing the STRING database [31]. A composite score exceeding 0.4 was deemed statistically significant when utilizing STRING to investigate interaction evidence. The findings were then imported into Cytoscape version 3.6.1, where a Bayesian network was created based on the selected candidate genes linked to HF [32].

### 2.7 Construction and verification of a diagnostic model of HF

In order to verify the accuracy of key genes, a model for diagnosing HF was developed by integrating these genes into a binary logistic regression framework utilizing the “proc” R package (1.18.5). Subsequently, this model was represented through a nomogram created with the “rms” R package (6.8−0). The effectiveness of the essential genes was assessed using Receiver Operating Characteristic (ROC) curves. To determine the diagnostic significance, the area under the curve (AUC) was calculated, where an AUC exceeding 0.7 is deemed representative of a suitable diagnostic marker.

### 2.8 Artificial neural network construction

A model using a Convolutional Neural Network (CNN) founded on MCP/gene scores was developed for the test groups GSE17800 and GSE57338 with the help of the “keras” package (2.15.0) in R software. The ROC was utilized to assess the predictive capability of the validation group’s GSE29819.

### 2.9 Gene set enrichment analysis of biomarkers

GSEA is an algorithm used to evaluate the distribution trend of genes in a predefined gene set in a gene table sorted by phenotype correlation to determine their contribution to phenotype [33]. We downloaded “c2.cp.kegg.v7.0” symbols.gmt datasets from the MSigDB database. The “clusterprofiler” R package (4.10.1) was utilized to perform GSEA on the co-expressed genes, aiming to explore the biological processes associated with these genes.

### 2.10 Immune infiltration analysis

We performed ssGSEA analysis on patients with a training set by means of the “GSVA” package (1.50.5) [34], which in turn estimated the composition and abundance of immune cells, and compared the differences in immune cells between the disease and the control group, as well as the correlations of key genes and immune cells.

### 2.11 Consensus clustering

Utilizing the expression profiles of key genes, the R package “ConsensusClusterPlus” (1.66.0) [24] was employed to identify distinct HF-related groups. The resulting clusters were subsequently analyzed by comparing their expression levels through heatmaps and box plots. PCA was performed to validate the clustering results.

### 2.12 Assessment of key genes expression on single-cell RNA-seq data

We used the R package “Seurat” (4.4.0) [35], and created the expression matrix of the GSE145154 data set as a Seurat object. The proportion of mitochondrial genes in all genetic material may indicate whether the cell is in a homeostatic state. We usually think that when a cell has a higher proportion of mitochondrial genes than all genes, it may be in a stressful state. Therefore, we filtered out cells with mitochondrial gene content >20%, cells with nCount > 30,000 and cells with nFeature > 5000. Next, we standardize the sequencing depth of the data set by the “NormalizeData” function, and we screen out 2000 hypervariable genes. Subsequently, PCA [36] was applied to identify significant principal components, and the P-value distribution was visualized using the Elbowplot function. By calling the “FindClusters” function, the cells were divided into 25 different subpopulations.

### 2.13 Pseudotime trajectory analysis and cell communication analysis

The “Monocle” R package (2.24.0) [37] was employed to examine the pseudotime trajectory of fibroblasts. Commonly used to analyze cell-to-cell communication networks from single-cell transcriptome sequencing data, in order to further explore the role that genes play in HF fibroblasts, we used the “CellChat R package” (1.6.1) [38] to quantitatively infer and analyze cell-cell communication networks from single-cell RNA sequencing data, showed the interaction relationships of cell populations using circle diagrams, and used bubble diagrams to count all important receptor ligand pairs during cell-cell communication [39].

### 2.14 Drug–Protein interaction and molecular docking analysis of biomarkers

Based on the functional analysis of the four selected hub genes, we further explored potential targeted therapeutic agents. Protein-coding genes with therapeutic relevance were prioritized, and drug selection was restricted to compounds with established or potential cardioprotective effects. Initially, a catalog of small-molecule compounds interacting with the target genes was retrieved from the Comparative Toxicogenomics Database (CTD, https://ctdbase.org/) (S1 Table). Molecular structures of pirinixic acid and resveratrol were obtained from the PubChem database (https://pubchem.ncbi.nlm.nih.gov) [40].

To evaluate binding affinities and interaction patterns between candidate compounds and their corresponding protein targets, molecular docking was performed using AutoDock Vina (1.2.2) [41]. The 3D protein structures of ITIH5 (UniProt ID: Q86UX2) and ISLR (UniProt ID: O14498) were retrieved from the AlphaFold Protein Structure Database (https://alphafold.ebi.ac.uk/).

Prior to docking, all protein and ligand structures were converted into PDBQT format. Water molecules were removed, and polar hydrogen atoms were added. The docking grid box was defined to encompass the relevant binding domains, with dimensions set to 126Å × 100Å × 100Å and a grid spacing of 1 nm. The protein targets were considered rigid during docking, while the ligands were treated as flexible. Rotatable bonds in each compound were defined using AutoDock Tools to enable flexible conformational sampling. Molecular docking visualizations were performed using PyMOL 2.2.0.

## 3 Results

### 3.1 Differential expression analysis

**Fig 2:**
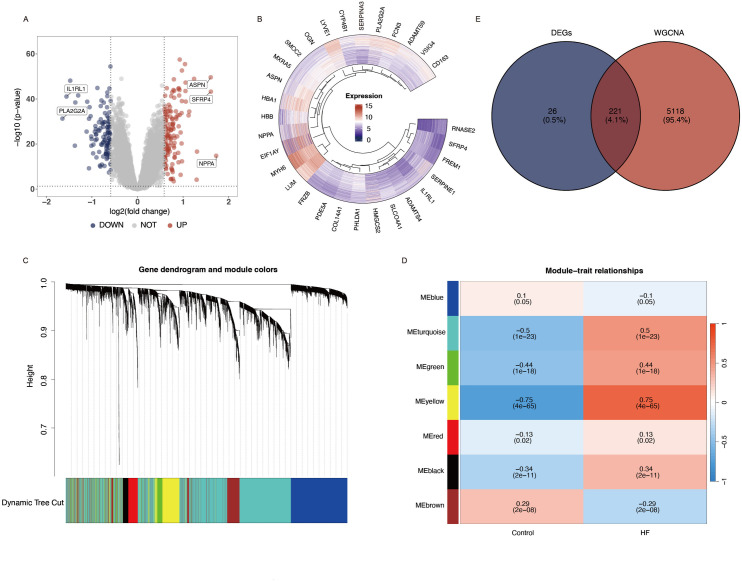
Screening differentially expressed genes in heart failure. **(A)** Volcano plot of DEGs. Red dots represent up-regulated genes and blue dots represent downregulated genes. **(B)** Differential gene expression heatmap. **(C)** Dendrogram of all genes with dissimilarity clustered on topological overlap. **(D)** The correlation heatmap representing the relationship between different gene modules and status of HF. **(E)** The venn diagram showing the intersection of DEGs with WGCNA.

The gene expression matrices were acquired, which included 144 control samples and 217 HF disease samples. A two-dimensional PCA clustering diagram was employed to visualize the integrated gene expression matrix from the two datasets, both prior to and following the batch effect removal. The batch effects in the two HF gene datasets are evident, all samples in the dataset achieved acceptable homogeneity following PCA analysis (S1 Fig A and B). A total of 247 DEGs were identified, comprising 134 genes that were up-regulated and 113 that were down-regulated. The results of the DEGs are depicted in a volcano plot (Fig 2A) and heatmap (Fig 2B).

### 3.2 Construction of Co-expressed gene modules

To identify the primary modules closely linked to HF, we performed a WGCNA on the final integrated dataset employing the “WGCNA” R package. The analysis indicated that with a β value of 4, the resulting network closely resembled a scale-free topology. We identified six distinct modules, designated as turquoise, yellow, red, blue, green, brown, and black, respectively, by applying the dynamic branch cutting approach (Fig 2C). Heatmaps illustrating module-trait correlations showed a strong association between HF and the turquoise (correlation coefficient = 0.5, P = 1e-23), yellow (correlation coefficient = 0.75, P = 4e-65), and green (correlation coefficient = 0.44, P = 1e-18) modules (Fig 2D). In total, these modules encompassed 5,339 genes. Through the convergence of data from the volcano plot, heatmap, and WGCNA method, we identified 221 significant genes that were deemed critical in the context of HF and selected for further investigation (Fig 2E). Additionally, we constructed functional analysis proteomaps for HF. Polygons represent each KEGG pathway, with their sizes proportional to the protein ratio in each pathway (S1 Fig C).

### 3.3 Functional enrichment analysis

Enrichment analyses for GO and KEGG were conducted on 221 essential genes to clarify the common biological mechanisms associated with HF. The GO enrichment analysis categories comprised biological processes (BP), cellular components (CC), and molecular functions (MF). Notably, BP encompassed the organization of external encapsulating structures, extracellular matrix, and extracellular structures, as well as processes related to muscle systems, cell-substrate adhesion, and organic anions. CC focused on components such as the collagen-containing extracellular matrix, external side of the plasma membrane, apical region of the cell, neuronal cell body and cytoplasmic vesicle lumen. For MF, key functions included the structural constituents of the extracellular matrix, binding to glycosaminoglycans, sulfur compounds, and heparin, integrin binding, and collagen binding. The KEGG enrichment analysis indicated that these genes were predominantly enriched in the Cytoskeleton in muscle cells, the Thyroid hormone signaling pathway, the cGMP − PKG signaling pathway, and ECM−receptor interactions (S1 Fig D and E).

### 3.4 Identifying key genes by machine learning

**Fig 3:**
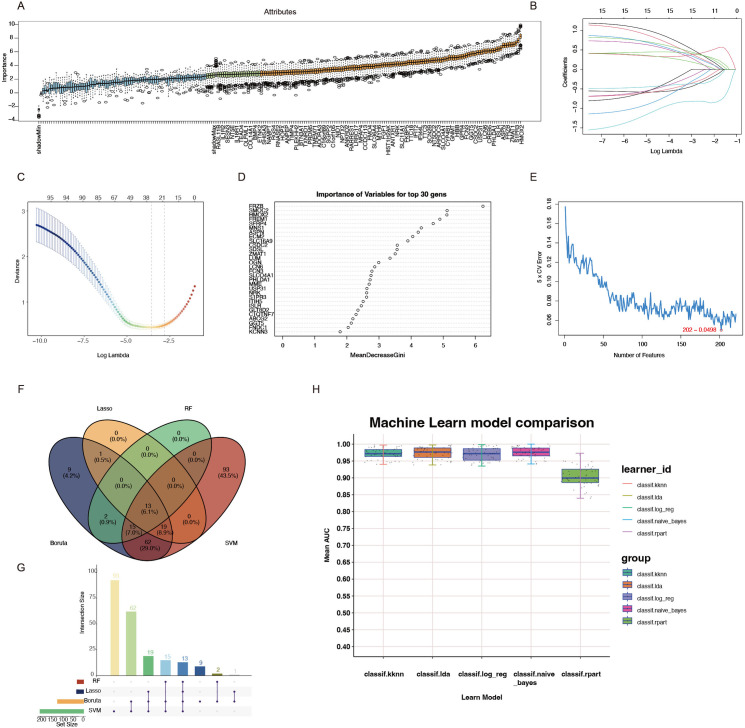
Multiple machine learning methods for screening heart failure related diagnostic biomarkers. **(A)** Boruta method identified biomarkers in HF datasets. **(B-C)** Screening for biomarkers using the Lasso model. **(D)** Results of the Gini coefficient method in random forest classifier. **(E)** SVM-RFE method identified biomarkers in HF datasets. **(F-G)** Intersection of genes selected by the four machine learning algorithms. **(H)** AUC comparison of five machine learning models.

In this study, we employed four unique machine learning algorithms, including LASSO, RF, Boruta, and SVM-RFE, to pinpoint critical genes linked to HF. The LASSO algorithm identified 33 potential candidate genes associated with HF. Using the RF algorithm, which evaluates genes according to their significance scores, we determined 30 leading candidate genes. The Boruta method uncovered 121 potential candidate genes. Likewise, SVM-RFE demonstrated optimal accuracy with a set of 202 genes, which were subsequently selected as candidates (Fig 3A-3E). By intersecting the findings from all four algorithms, we identified 13 essential genes for HF, comprising 8 up-regulated and 5 down-regulated genes (Fig 3F and 3G). The important genes underwent further validation through various machine learning techniques, and their diagnostic capabilities were evaluated by calculating the AUC. The AUC values derived from the ROC curves were found to be 0.93495–0.99839 for log_reg, 0.93825–0.99750 for lda, 0.94025–0.99719 for kknn, 0.941222−1 for naive_bayes, and 0.83973–0.97300 for rpart (Fig 3H).

Concurrently, the box plot illustrated the expression variations of core genes in normal samples compared to HF samples (S1 Fig F). Ultimately, we selected the 8 upregulated genes for further construction of the PPI network. Furthermore, to minimize potential sampling bias, we analyzed the correlations between the candidate key genes (S1 Fig G).

### 3.5 PPI network construction and key gene selection

**Fig 4:**
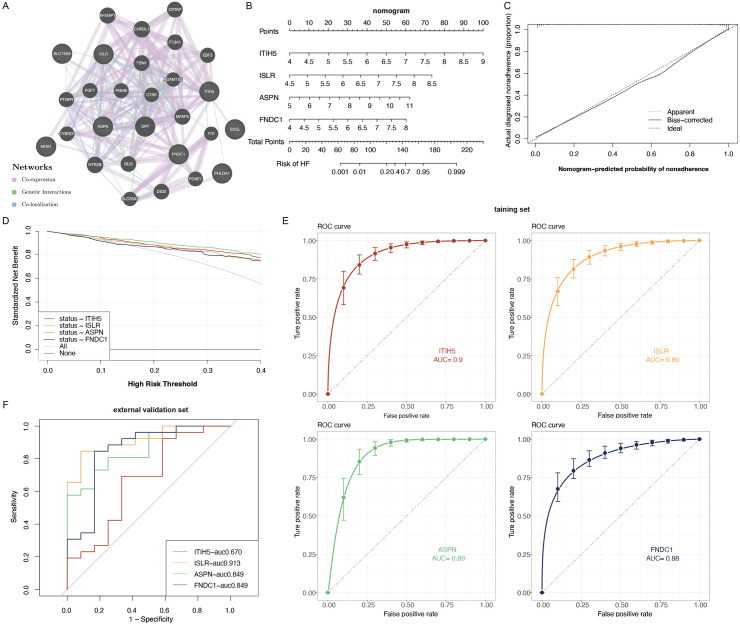
Validation of the diagnostic efficacy of characteristic genes. **(A)** PPI network for the obtained 8 key genes constructed by GeneMANIA. Edge colors represent interactions based on co-expression, physical interactions, co-localization, shared protein domains, or predicted interactions. **(B)** Nomogram predicting patient survival prognosis. **(C)** Calibration curve to examine the predictive power of the diagnostic column line graph. **(D)** DCA to evaluate the predictive power of the diagnostic column line graph. **(E)** ROC curves of the diagnostic sensitivity and specificity of each gene individually as well as the nomogram. **(F)** The diagnostic efficacy of the signature genes was further validated through the application of an external dataset.

8 genes related to HF entered into the STRING database to explore the functional connections between protein-coding genes, 20 related genes were shown. In the end, we discovered 28 nodes and 298 interactions in the network. Notably, ITIH5, ISLR, ASPN and FNDC1 demonstrated significant interaction relationships related to HF. As a result, we chose four key genes, including ITIH5, ISLR, ASPN, and FNDC1 (Fig 4A). To further clarify the chromosomal distribution of the identified genes, the co-expressed genes were visualized (S1 Fig H).

### 3.6 Diagnostic value assessment

Nomograms based on the four crucial genes were developed, and ROC curves were used to evaluate the diagnostic performance of each gene and the nomogram. Within the nomogram, each signature variable was allocated a particular score, and the overall score was computed by aggregating all the feature variables, reflecting the likelihood of HF occurrence (Fig 4B). The calibration curve validated the nomogram’s capability in accurately diagnosing HF (Fig 4C). Moreover, Decision Curve Analysis (DCA) revealed that employing the nomogram offered substantial clinical benefits for patients suffering from HF (Fig 4D). In the training dataset, the AUC values for the ROC curves were determined to be 0.9 for ITIH5, 0.89 for ISLR, 0.89 for ASPN, and 0.88 for FNDC1 (Fig 4E). Additionally, the diagnostic efficacy of the four signature genes was further validated through the application of an external dataset (GSE29819) (Fig 4F). In summary, the identified key genes exhibited superior predictive ability regarding the progression of HF.

### 3.7 Deep learning CNN model differentiates HF and non-HF patients using gene expression and cell type associations

**Fig 5:**
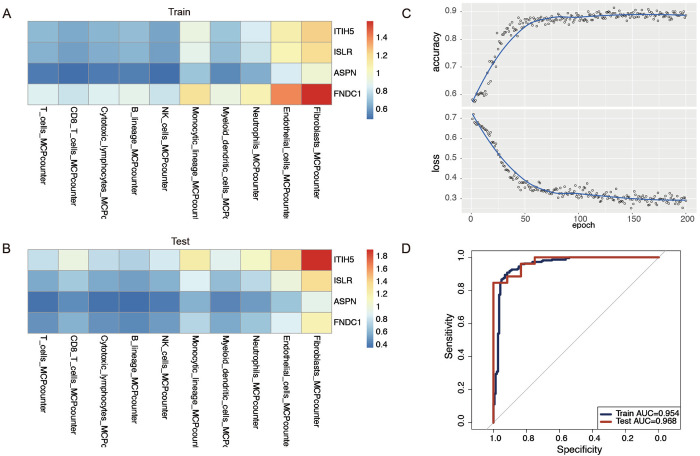
Creating a deep learning model utilizing artificial convolutional neural networks for the purpose of distinguishing between HF and non-HF patients. **(A)** Heatmap cards of patients in the training set, made based on mcpcounter scores expression levels. **(B)** Heatmap cards of patients in the validation set, made based on HF expression levels. **(C)** Training process of the convolutional neural network. **(D)** AUC for diagnostic performance in the training set and validation set.

Using MCPcounter, various scores for cell types were calculated for both cohorts. Subsequently, the expression levels of key genes were assessed across various cell types, including immune cells, fibroblasts, and epithelial cells, for each individual in both the training and validation cohorts. A two-dimensional array was generated for each individual to depict the relationships between these essential genes and various cell types, with the findings presented as heatmaps (Fig 5A and 5B). The training set comprised the GSE17800 and GSE57338, whereas the GSE29819 acted as the validation dataset. The training process was executed for 200 iterations to enhance accuracy and mitigate bias (Fig 5C). The ROC curves for the training and validation sets were recorded at 0.954 and 0.883, respectively, indicating that the deep learning CNN model possesses strong sensitivity and accuracy in distinguishing between HF and non-HF patients (Fig 5D).

### 3.8 GSEA enrichment analysis

**Fig 6:**
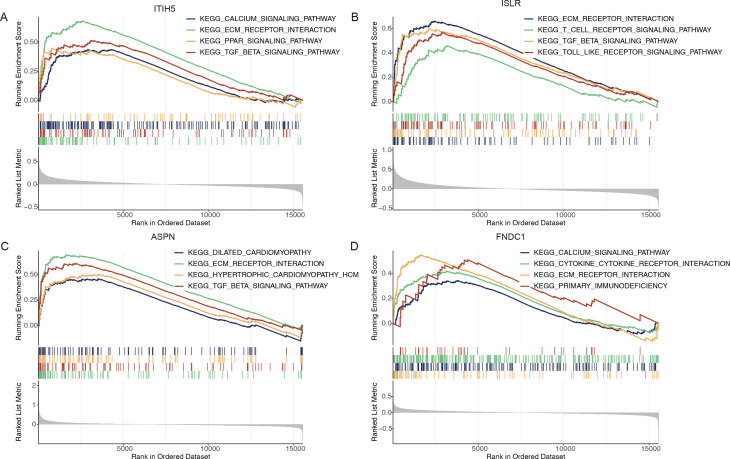
The results of ssGSEA enrichment analysis. **(A)** The results of ITIH5. **(B)** The results of ISLR. **(C)** The results of ASPN. **(D)** The results of FNDC1.

To further investigate the possible links between diagnostic markers and immune responses in the development of HF, GSEA analysis was performed. The results indicated that the immune-related pathways enriched for ITIH5 were primarily associated with the Calcium signaling pathway, TGF-β signaling pathway, ECM receptor interaction, and PPAR signaling pathway (Fig 6A). In the case of ISLR, it was predominantly linked to the ECM receptor interaction, T cell receptor signaling pathway, TGF-β signaling pathway, and Toll-like receptor signaling pathway (Fig 6B). Regarding ASPN, the main associations were with Dilated Cardiomyopathy (DCM), ECM receptor interaction, Hypertrophic Cardiomyopathy (HCM), and the TGF-β signaling pathway (Fig 6C). For FNDC1, significant involvement was observed in the Calcium signaling pathway, Cytokine-Cytokine Receptor Interaction, and Primary Immunodeficiency (Fig 6D).

### 3.9 Immune cell infiltration analysis

To examine the involvement of immune cells in the advancement of HF, we conducted an analysis of immune cell infiltration across both HF datasets utilizing the CIBERSORT algorithm. Significant differences were observed in specific immune cell subtypes, including Activated CD4 T cell, Effector memory CD4 T cell, Activated CD8 T cell, Type 2 T helper cell, Central memory CD8 T cell and Natural killer T cell, when comparing samples from the HF group to control samples. In addition, Analysis conducted on 28 types of immune cells reveals that the co-expressed genes ITIH5, ISLR, ASPN, FNDC1 show a positive association with Activated CD4 T cell, Activated CD8 T cell, Effector memory CD4 T cell and Type 2 T helper cell. while they exhibit a negative correlation with Central memory CD8 T cell and Natural killer T cell (S2 Fig).

### 3.10 Unsupervised cluster analysis of the key genes in HF

To investigate the essential genes associated with HF, we carried out an unsupervised consensus clustering analysis of HF samples, utilizing the expression profiles of these critical genes. The clustering results revealed that k = 2 was the most appropriate choice, with HF patients being accurately categorized into two distinct groups (S3 Fig A and B). Gene expression in cluster 2 was found to be greater compared to that in cluster 1 (S3 Fig C). Based on the limited clinical data at hand, there were no notable differences in terms of age or gender between the two groups (S3 Fig D).

### 3.11 Normalization of scRNA-seq data

In the GSE145154 dataset, several low-quality samples were excluded. Ultimately, a total of 15,004 cells from HF patients and 18,730 cells from the control group were included for subsequent analysis. The visualization of gene quantity, sequencing depth, and mitochondrial gene content confirmed the quality and availability of the scRNA data (S4 Fig A-C). The percentage of mitochondrial genes was independent of the gene count, while sequencing depth showed a positive correlation with gene count, with a coefficient of 0.92 in the HF group (S4 Fig D).

### 3.12 Identification of clusters and cell types

**Fig 7:**
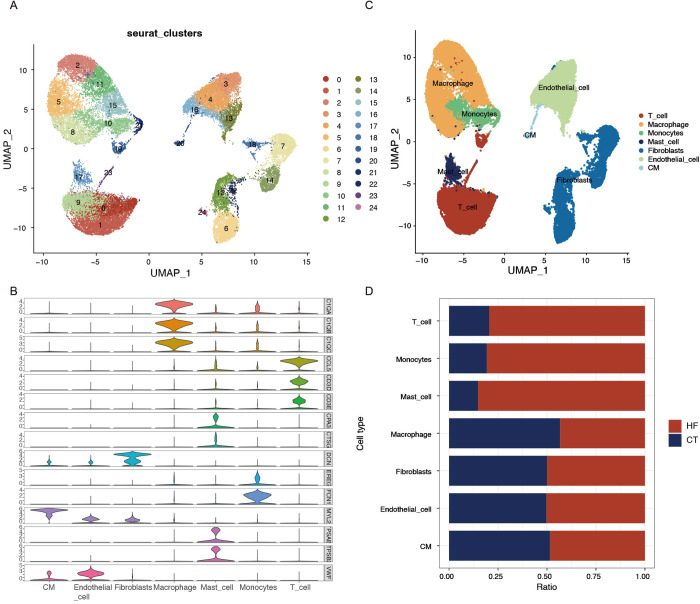
Overview of the 33,734 single cells from HF and control samples. **(A)** Clustering UMAP plots of different clusters of cells in HF samples. **(B)** Violin plot showing the expression of signaling genes. **(C)** UMAP plots of different subclusters of cells in HF samples. **(D)** Proportional representation of different cell types in HF and control samples.

UMAP analysis divided the cell population into 25 clusters (Fig 7A). Each cluster was annotated based on the classical cell marker, annotating the cells into 7 large subpopulations, including T-cells, mast cells, CM, macrophages, endothelial cells, fibroblasts, and monocytes (Fig 7B-7C). And the distribution of the proportion of cells of these cells is shown (Fig 7D). Subsequently, the research concentrated on the localization of key genes (ASPN, ITIH5, ISLR, and FNDC1) within UAMP plots. According to the UAMP analysis, fibroblasts showed a marked expression of ASPN, ITIH5, and ISLR (S4 Fig E-H).

### 3.13 Pseudotime trajectory analysis based on key genes

**Fig 8:**
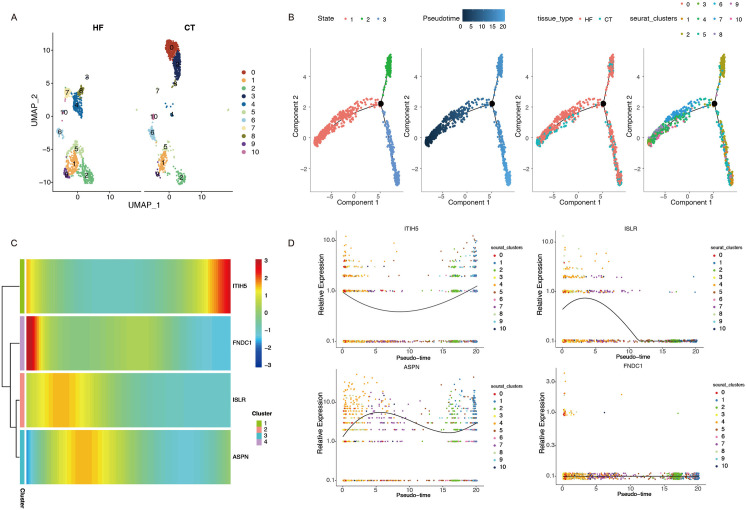
Trajectory analyses of a single cell. **(A)** Clustering UMAP plots of fibroblasts in HF and control samples. **(B)** Trajectory analyses of a single cell. **(C)** Heatmap depicting ITIH5, FNDC1,ISLR and ASPN in fibroblasts based on the pseudotime trajectory. **(D)** Dynamic expression of ITIH5, ISLR, ASPN and FNDC1 within pseudotime.

To investigate the dynamic transitions of cells and gene expression changes during disease progression, we performed pseudotime trajectory analysis. Fibroblasts were grouped into nine clusters using UMAP, based on both HF and control samples (Fig 8A). Cells were arranged along developmental trajectories, revealing distinct shifts in cellular states between the HF and control groups (Fig 8B). ITIH5 and ASPN showed increasing expression trends along pseudotime, suggesting their association with fibroblast activation and fibrosis-related processes (Fig 8C-8D).

### 3.14 Cell communication analysis

**Fig 9:**
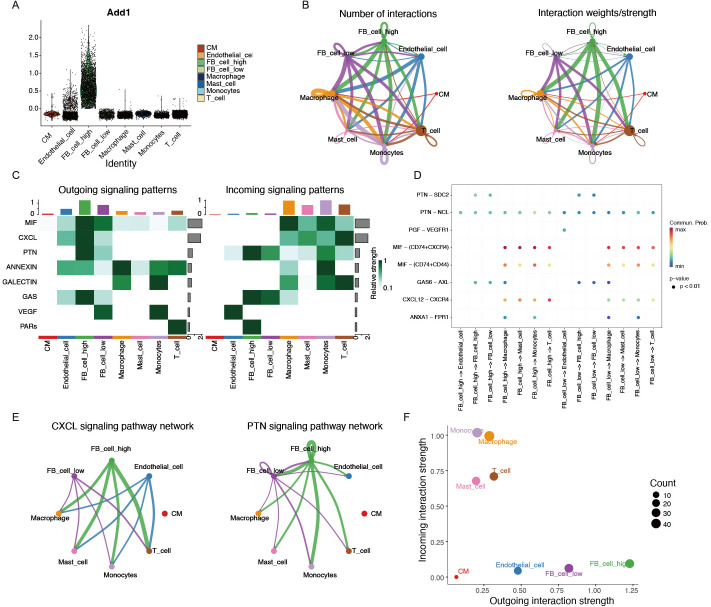
Comprehensive CellChat analysis of all cell types. **(A)** The violin plot shows the four key genes are based on expression scores in fibroblasts. **(B)** Communication interactions network plot for all cell types. **(C)** Heatmap showing the contribution of incoming and outgoing signals for each cell type. The bar at the top indicates the proportion of each cell type’s ligands. **(D)** Bubble diagram of all potential ligand-receptor interactions between fibroblasts and other cell types. The x-axis shows the interacting cell types, the y-axis shows ligand-receptor pairs. Dot color indicates interaction likelihood, and dot size represents corresponding P-values. **(E)** Overview of CXCL and PTN signalings networks. **(F)** Outgoing and incoming interaction strength of all cell types.

To explore the functional roles of four key genes in HF fibroblasts, we scored fibroblasts based on the expression of these genes and classified them into two subpopulations: high-scoring fibroblasts and low-scoring fibroblasts (Fig 9A). Both outgoing and incoming signaling strength analyses revealed that fibroblasts-particularly the FB_cell_high subgroup-acted as a central signaling hub, transmitting signals to multiple immune and structural cell types (Fig 9B and 9C). Notably, several ligand-receptor pairs were enriched in HF samples, including PTN-NCL and CXCL12-CXCR4, which were predominantly mediated by high-scoring fibroblasts (Fig 9D). The signaling networks of CXCL and PTN pathways were particularly active in HF, underscoring the importance of fibroblast-derived paracrine communication (Fig 9E). Finally, we summarized the overall interaction strengths across different cell types (Fig 9F).

### 3.15 Drug–Gene interaction and molecular docking analysis of biomarkers

**Fig 10:**
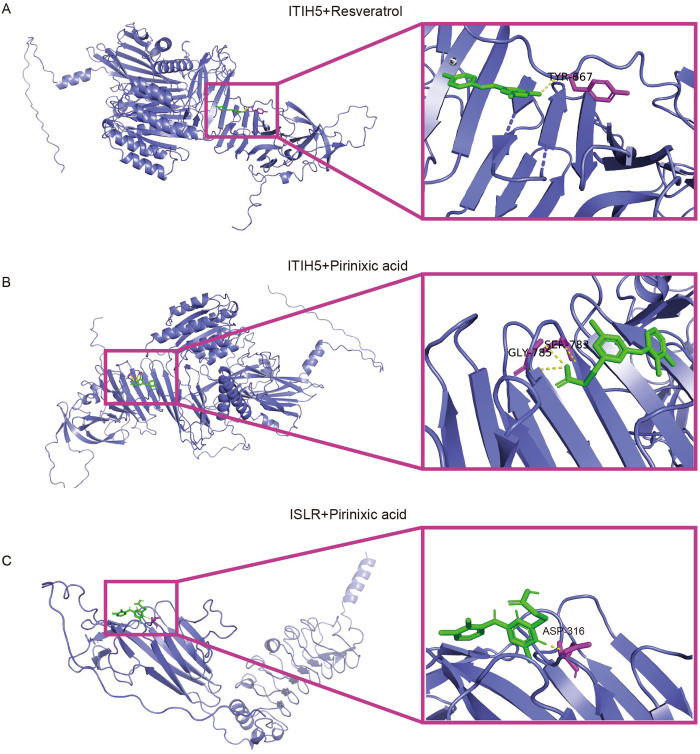
The docking results of diagnostic genes encoded proteins with small molecular compounds. **(A)** Resveratrol interacts with the amino acid residue TYR-667 of ITIH5. **(B)** Pirinixic acid interacts with the amino acid residue GLY-785 and SER-783 of ITIH5. **(C)** Pirinixic acid interacts with the amino acid residue ASP-316 of ISLR.

We next utilized the CTD database, drug toxicology studies and auto molecular docking to explore the drugs targeted to diagnostic genes. Since ASPN and FDN1 currently lack corresponding cardioprotective drugs, we selected ITIH5 and ISLR for molecular docking with resveratrol and pirinixic acid, respectively. To evaluate whether the proteins encoded by these clinically relevant feature genes could effectively bind to the selected compounds, we performed molecular docking analysis. We found that the majority of docking combinations had binding energy lower than −5.0 kcal/mol through molecular docking analysis of the feature genes, the lower docking score indicates a stronger binding affinity [42]. The best docking combinations are shown. Notably, resveratrol showed a strong binding affinity with residues TYR-667 of ITIH5 through hydrogen bonding, with a docking energy of −6.98 kcal/mol. Additionally, pirinixic acid interacted with ITIH5 through hydrogen bonding, involving GLY-785 and SER-783, with a docking energy of −6.93 kcal/mol. Moreover, pirinixic acid was found to bind to residues ASP-316 of ISLR through hydrogen bonding, with a docking energy of −3.28 kcal/mol (S2 Table). The molecular docking visualizations were conducted utilizing PyMOL 2.2.0 software (Fig 10A-10C). The specific structures of two key genes with two compounds were shown (S5 Fig). However, it is important to note that molecular docking provides only theoretical predictions of binding affinity and interaction patterns. Further experimental validation, such as in vitro binding assays, cell-based functional studies, or in vivo experiments, is required to confirm the biological relevance and therapeutic potential of these predicted interactions.

## 4 Discussion

HF has become a rising public health challenge, marked by chronic cardiac dysfunction stemming from a variety of etiologies. Patients with HF experience a range of debilitating symptoms that significantly impair their quality of life [43,44]. In clinical practice, BNP remains the gold standard biomarker for diagnosing and assessing HF. Nonetheless, recent research has revealed certain limitations, as extremely low levels of BNP (<50 pg/ml) were noted in 4.9% of patients with HF, and a minor percentage (ranging from 0.1% to 1.1%) exhibited BNP levels that fell beneath detection thresholds [45]. These findings underscore the need for exploring additional molecular mechanisms underlying HF to improve diagnosis and therapeutic approaches. This highlights the necessity for additional research into the molecular mechanisms that drive HF to alleviate its increasing impact. The advancement and progression of HF encompass a complex interaction of biological processes, featuring inflammatory responses, oxidative stress, and neuroendocrine activation. Discovering new molecular biomarkers is essential for enhancing our comprehension of HF pathogenesis. The advent of high-throughput technologies and bioinformatics tools has improved the efficiency of screening potential biomarkers and therapeutic targets [46]. Additionally, the application of machine learning models in bioinformatics has shown promise in uncovering underlying mechanisms, enhancing diagnostic capabilities, and identifying new therapeutic avenues for HF [47].

To systematically uncover molecular drivers of HF pathogenesis, we first identified 221 DEGs in HF through the intersection of volcano plots, heatmaps, and WGCNA methods. Building on these DEGs, functional enrichment analyses revealed that HF progression is predominantly governed by extracellular matrix (ECM) remodeling, collagen dynamics, and dysregulation of the cGMP-PKG signaling pathway. Notably, ECM-receptor interactions emerged as a central hub, corroborating the dual role of ECM in maintaining cardiac structural integrity and transmitting pathological mechanical stress—a hallmark of HF initiation and progression [48]. This mechanistic link was further reinforced by the cGMP-PKG pathway, which modulates vascular tone and fibrosis, possibly via crosstalk with fibroblasts, cardiomyocytes, and immune cells [49], positioning it as a therapeutic target for HFpEF [50]. To prioritize clinically actionable biomarkers, using four machine learning algorithms, we further identified eight upregulated and five downregulated candidate genes in HF. Despite their initial identification as DEGs, BMP4 and NPPA were excluded from downstream models due to limited diagnostic utility, highlighting the necessity for robust feature selection. Subsequent PPI network analysis distilled four core genes—ITIH5, ISLR, ASPN, and FNDC1—whose diagnostic power was validated externally (training cohort AUC = 0.954; validation cohort AUC = 0.968) using CNN-based heatmap models [51,52]. This multi-algorithm approach provides a potential supplement to the limitations of BNP by identifying a novel gene panel for HF risk stratification. Considering the well-established role of inflammation in HF [53], we profiled immune cell infiltration across 28 subtypes. The results indicated that four immune cells—Activated CD8 T cells, Activated CD4 T cells, Effector memory CD4 T cells, and Type 2 T helper cells—were significantly more infiltrated in the HF group. These findings align with evidence that TNF-α-mediated suppression of cardiac-reactive CD4 + T cells mitigates myocardial injury [54], suggesting immunomodulation as a complementary therapeutic axis. To unify the roles of ITIH5, ISLR, ASPN, and FNDC1, GSEA highlighted their convergent regulation of ECM-receptor interactions. Mechanistically, excessive ECM deposition disrupts ventricular function and elevates mortality risk [55]. In addition, another common pathway of ITIH5, ISLR and ASPN is TGF-β signaling pathway, while TGF-β activation drives fibrosis—a self-reinforcing cycle central to HF progression [56]. By linking these genetic markers to the relevant pathways, our study elucidates their potential cooperative roles in ECM and TGF-β signaling pathway dysregulation.

As for ITIH5, this gene belongs to the Inter-a-trypsin inhibitor (ITI) protein family and is involved in blood circulation and ECM regulation. ITIH5 may influence cell migration and focal adhesion formation, potentially altering cardiac fibroblast function and ECM remodeling in HF [57]. As for ISLR, the ISLR gene was associated with the Toll-like receptor (TLR) signaling pathway, which plays a significant role in modulating inflammatory responses in HF [58]. In the context of coronary artery disease – a major risk factor for HF – TLR2/4 expression on monocytes is elevated during acute myocardial infarction (AMI) [59]. Preclinical AMI models indicate that TLR2/4 inhibition reduces inflammatory cell recruitment and infarct size [60], while TLR4 blockade attenuates cardiomyocyte oxidative stress [61]. These findings collectively highlight TLR signaling as a potential therapeutic target in ischemia-driven HF. Beyond inflammatory mechanisms, genetic cardiomyopathies represent another pivotal driver of HF. Concerning ASPN, it is associated with two pathways, including HCM and DCM, the two main causes of HF. Its overexpression exacerbates cardiac fibrosis and systolic dysfunction, indicating its potential role in undesirable remodeling. Regarding FNDC1, this gene was linked to both ECM-receptor interactions and calcium signaling – pathways integral to myocardial structure and contractility. Calcium acts as a key second messenger involved in mitochondrial function and ATP production. Mitochondrial dysfunction and metabolic remodeling are central to the pathogenesis of HF [62], indicating that FNDC1 might contribute to the energetic regulation of cardiac cells. In addition, FNDC1 facilitates vascular endothelial growth factor-mediated angiogenesis [63,64]. Notably, a study proposed serum FNDC1 as a candidate HF biomarker [65]. This dual role in both mechanistic regulation and diagnostic potential exemplifies how bridging molecular pathways to clinical applications could advance precision medicine in HF management.

To elucidate the cellular origins and transcriptional heterogeneity of these core genes, we employed scRNA-seq. Notably, fibroblasts exhibited marked expansion in HF samples—a finding aligned with prior evidence that pathological fibroblast activation drives maladaptive fibrosis, arrhythmogenesis, and HF decompensation [66]. Pseudotime trajectory analysis further delineated a progressive upregulation of ITIH5 and ASPN during fibroblast differentiation, suggesting their synergistic role in orchestrating pro-fibrotic phenotypic switching. Moreover, cell-cell communication analysis suggested fibroblasts, particularly the FB_cell_high subgroup, as prominent contributors to HF microenvironment signaling. Prominent interactions included PTN and CXCL pathways. There are studies that show that under cellular stress conditions, the PTN pathway enhances cardiomyocyte apoptosis [67], and the CXCR4 pathway contributes to the pathogenesis of cardiac fibrosis in DCM and it may represent a new potential therapeutic target for HF [68]. Collectively, PTN-NCL and CXCL12-CXCR4 pathways were significantly upregulated, suggesting fibroblasts may play a role in myocardial injury and adverse remodeling.

To explore therapeutic compounds targeting the identified pathways, we screened the CDB database and prioritized candidates based on mechanistic relevance and clinical feasibility. The candidates included antitumor agents (Doxorubicin, Sodium arsenite, Thioguanine, Cyclosporine) and cardioprotective compounds (Pirinixic acid, Resveratrol). While antitumor agents theoretically align with cell cycle and apoptosis regulation in HF, their clinical application is limited by dose-dependent cardiotoxicity. In contrast, cardioprotective compounds may demonstrate a more favorable mechanistic alignment with HF pathophysiology. For example, pirinixic acid activates PPARα, a pathway critical for fatty acid metabolism. Previous studies in inducible transgenic mouse models revealed that PPARα activation preserves myocardial energetics and delays HF progression [69,70], highlighting its potential as a metabolic modulator. Similarly, resveratrol, a SIRT1 activator, mitigates oxidative stress and apoptosis in preclinical HF models. A recent study further demonstrated that resveratrol improves cardiac function via FoxO3a activation, suggesting SIRT1 as a viable target [71,72]. Our Resveratrol and Pirinixic acid exhibited binding energies of −6.98 kcal/mol and −6.93 kcal/mol, respectively, which are comparable to HF used clinically. For example, there is a study that shows that the binding energy of enalapril to the TNF molecule docking was −7.1 kcal/mol [73]. The lowest energies for Sacubitril-PPARα and Valsartan-PPARα interaction were −7.7 kcal/mol and −7.3 kcal/mol respectively [74]. Nevertheless, further molecular dynamics and experiments are needed in the future to verify its therapeutic effect.

It must be acknowledged that our study has several limitations. First, this study is limited by the lack of experimental validation for the predicted drug-gene interactions. Molecular docking simulations provide only theoretical predictions based on static protein structures and may not fully capture the dynamic nature of protein-ligand interactions in biological systems. Future studies involving in vitro and in vivo assays, as well as molecular dynamics simulations, are warranted to validate the therapeutic potential of the identified compounds and further elucidate their mechanisms of action in cardiovascular disease models. Second, our findings have not yet been validated in vivo or in vitro due to the absence of experimental conditions for follow-up validation. Third, computational methods such as machine learning, molecular docking, and scRNA-seq data analysis carry inherent limitations, including potential false positives arising from algorithmic biases or dataset-specific noise. Therefore, while our approach provides a prioritization framework, translational applications demand rigorous experimental verification.

Despite these limitations, our integrated bioinformatics approach systematically identified potential candidate genes and therapeutic compounds, providing a foundation for further mechanistic exploration in HF pathogenesis. In conclusion, this analysis highlights putative diagnostic genes, suggests potential molecular mechanisms, and proposes candidate therapeutic compounds. These findings offer valuable insights into HF pathogenesis, yet further validation through experimental studies and clinical trials is warranted.

## 5 Conclusions

In this study, we employed an integrated bioinformatics approach to identify key diagnostic genes and potential therapeutic compounds for HF. Specifically, we identified four candidate diagnostic genes (ASPN, ITIH5, ISLR, and FNDC1) associated with fibroblast activation and myocardial remodeling and two candidate compounds (pirinixic acid and resveratrol) targeting PPAR and SIRT1 pathways, respectively. These findings reinforce the critical roles of metabolic dysregulation, oxidative stress, and fibroblast-driven fibrosis in HF pathogenesis and offer promising avenues for risk stratification and targeted therapy. Future studies should focus on validating the diagnostic and therapeutic potential of these targets and exploring their mechanisms in experimental models.

## Supporting information

S1 Fig Enrichment Analysis and Gene Expression (A) PCA score plot of the training group dataset before batch correction. (B) PCA score plot of the training group dataset after batch correction. (C) Proteomap visualization. (D) KEGG enrichment analysis of candidate hub genes. (E) GO enrichment analysis of candidate hub genes. (F) The box plot of the expression variations of core genes in normal samples compared to HF samples. (G) The correlations analysis between the candidate key genes.(H) Circos track plot used to map the location of 4 shared genes on the chromosomes.
(TIF)

S2 Fig Analysis of Immune Cell Infiltration. (A) The distribution of 28 immunocytes between the HF and control samples. (B) The correlation heatmap showed the correlation between different immunocytes in HF samples. (C) The correlation of key genes and immune cells.
(TIF)

S3 Fig Identification of two distinct subtypes across HF samples. (A) Consensus matrix heatmap defining two subtypes (k = 2) and their correlation area. (B) PCA showing a remarkable difference between the two subtypes of HF. (C) The two subtypes exhibit distinct expression profiles. (D) Expression heatmap of HF-related genes in the two subtypes.
(TIF)

S4 Fig Gene Expression Distribution in Cell Types (A-C) Single-cell sequencing depth, counts and fraction of reads mapped to mitochondrial genes in HF groups. (D) Correlation of gene count of mitochondrial genes and features in HF. (E-H) Distribution of ITIH5, ISLR, ASPN and FNDC1 in each cell type.
(TIF)

S5 Fig Schematic diagram of the structure of small molecule proteins and compounds. (A) ITIH5 protein structure. (B) ISLR protein structure. (C) Resveratrol chemical Structure. (D) Pirinixic acid chemical structure.
(TIF)

S1 Table The details of the candidate small molecular drugs targeting key genes. (DOCX)

S2 Table Molecular docking binding energy of candidate small molecular drugs and targeting key genes. (DOCX)
