# Supplementary figures and images for: Uncovering key biomarkers, potential therapeutic targets and development of deep learning model in heart failure
Source: PLoS One. 2025 Sep 3;20(9):e0330780. doi: 10.1371/journal.pone.0330780 (PMC12407452; doi:10.1371/journal.pone.0330780)

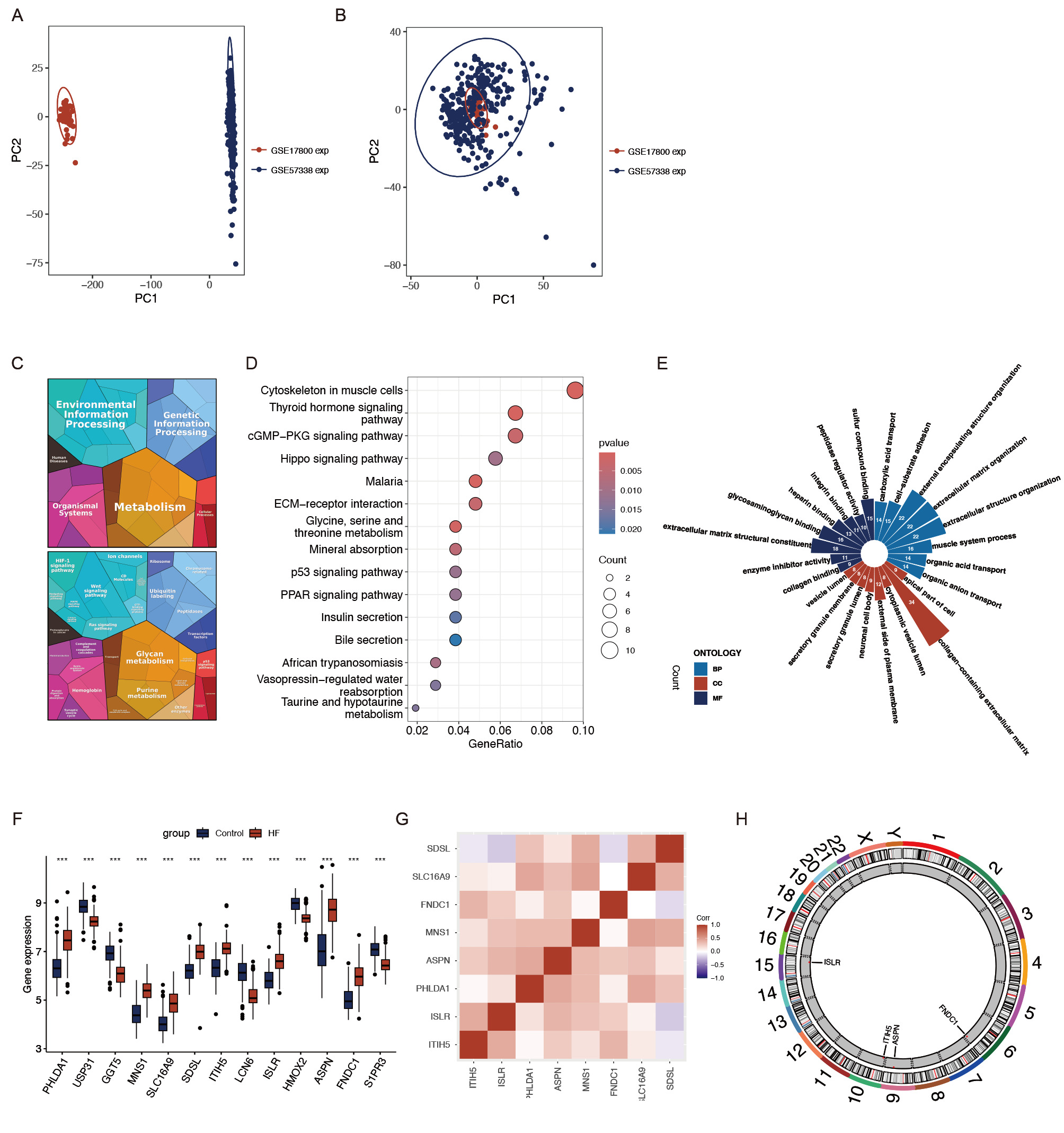

Supplement: S1 Fig — (B) PCA score plot of the training group dataset after batch correction. (C) Proteomap visualization. (D) KEGG enrichment analysis of candidate hub genes. (E) GO enrichment analysis of candidate hub genes. (F) The box plot of the expression variations of core genes in normal samples compared to HF samples. (G) The correlations analysis between the candidate key genes.(H) Circos track plot used to map the location of 4 shared genes on the chromosomes. (TIF) [file pone.0330780.s001.tif]

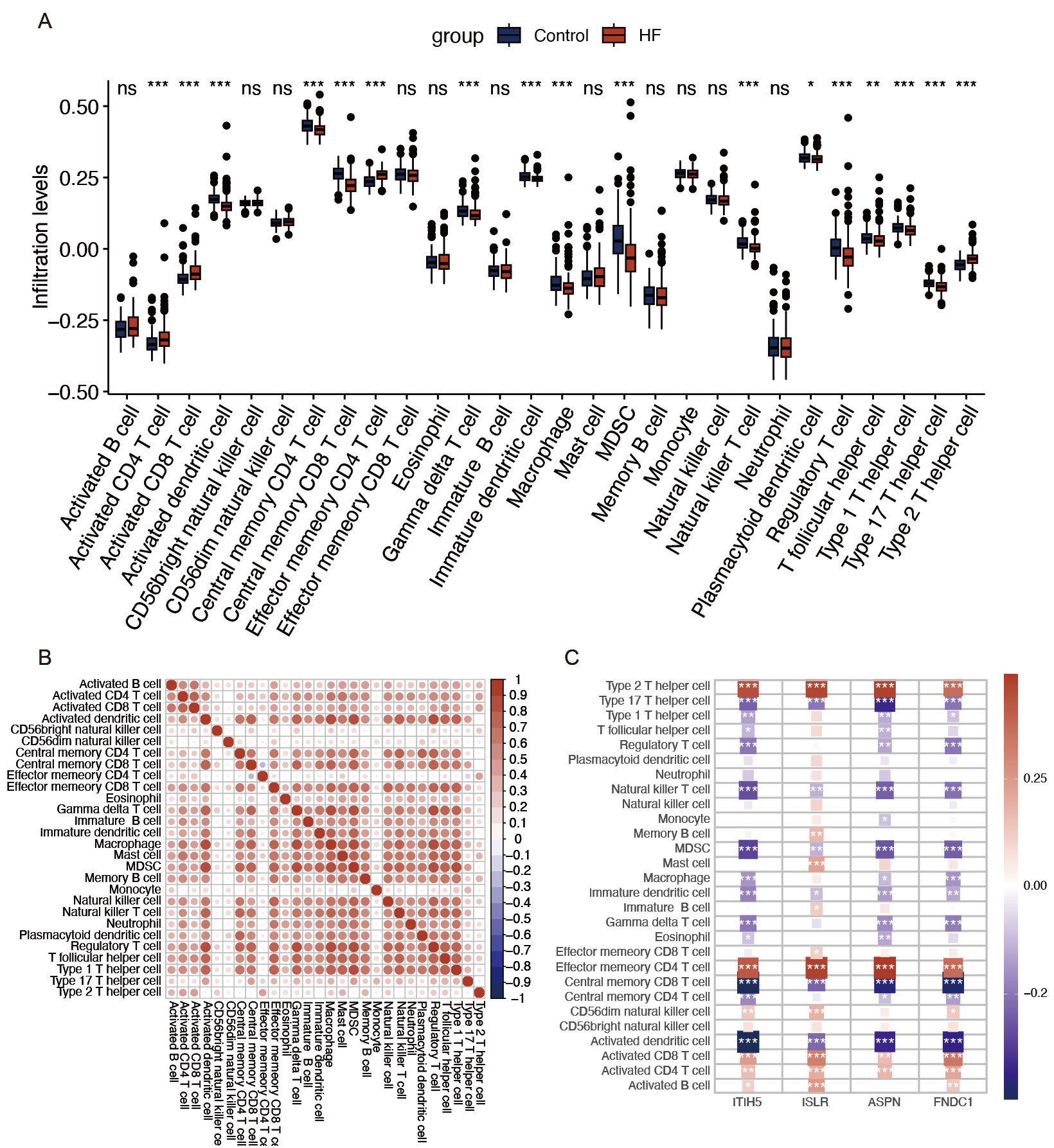

Supplement: S2 Fig — (A) The distribution of 28 immunocytes between the HF and control samples. (B) The correlation heatmap showed the correlation between different immunocytes in HF samples. (C) The correlation of key genes and immune cells. (TIF) [file pone.0330780.s002.tif]

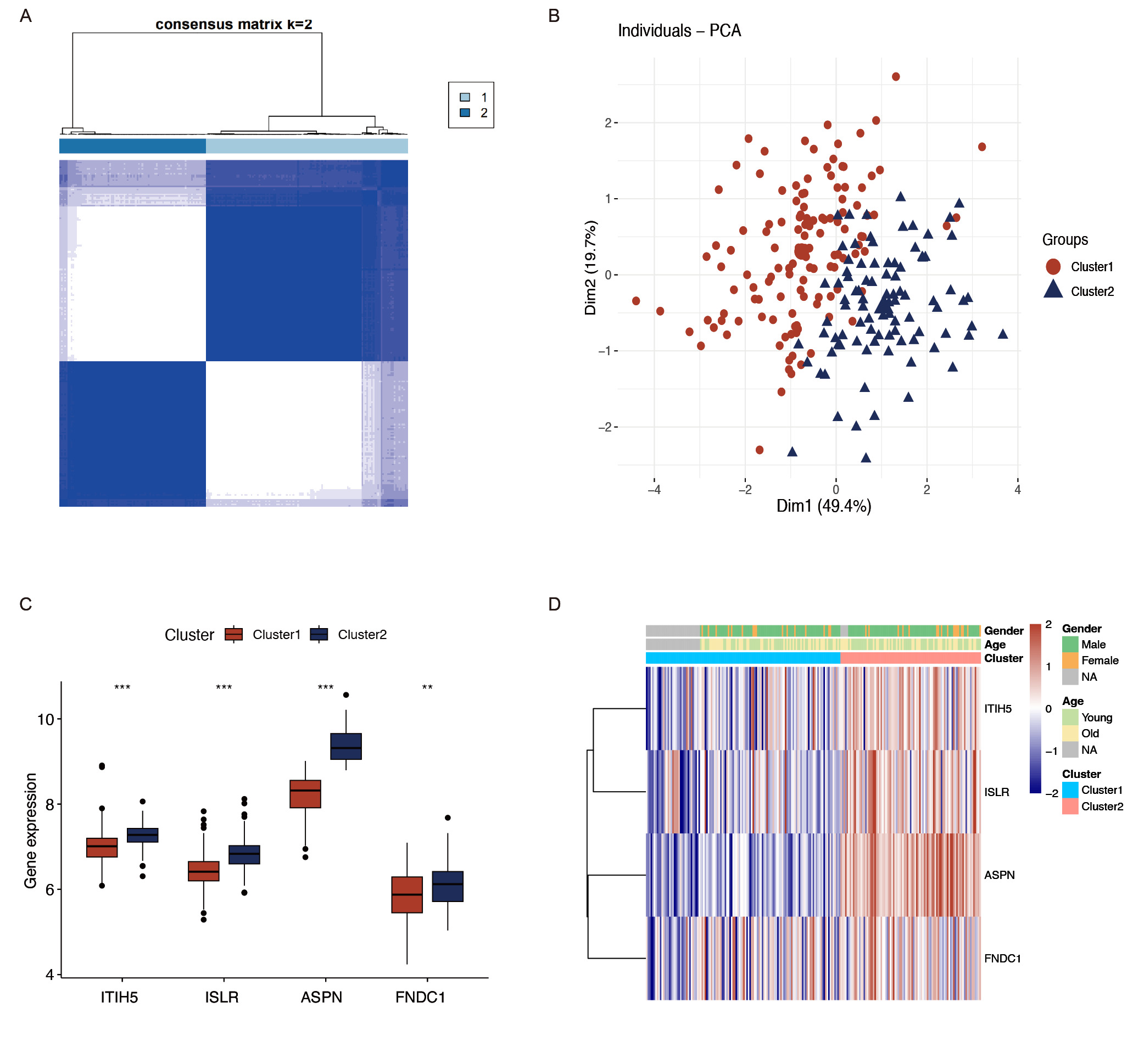

Supplement: S3 Fig — (A) Consensus matrix heatmap defining two subtypes (k = 2) and their correlation area. (B) PCA showing a remarkable difference between the two subtypes of HF. (C) The two subtypes exhibit distinct expression profiles. (D) Expression heatmap of HF-related genes in the two subtypes. (TIF) [file pone.0330780.s003.tif]

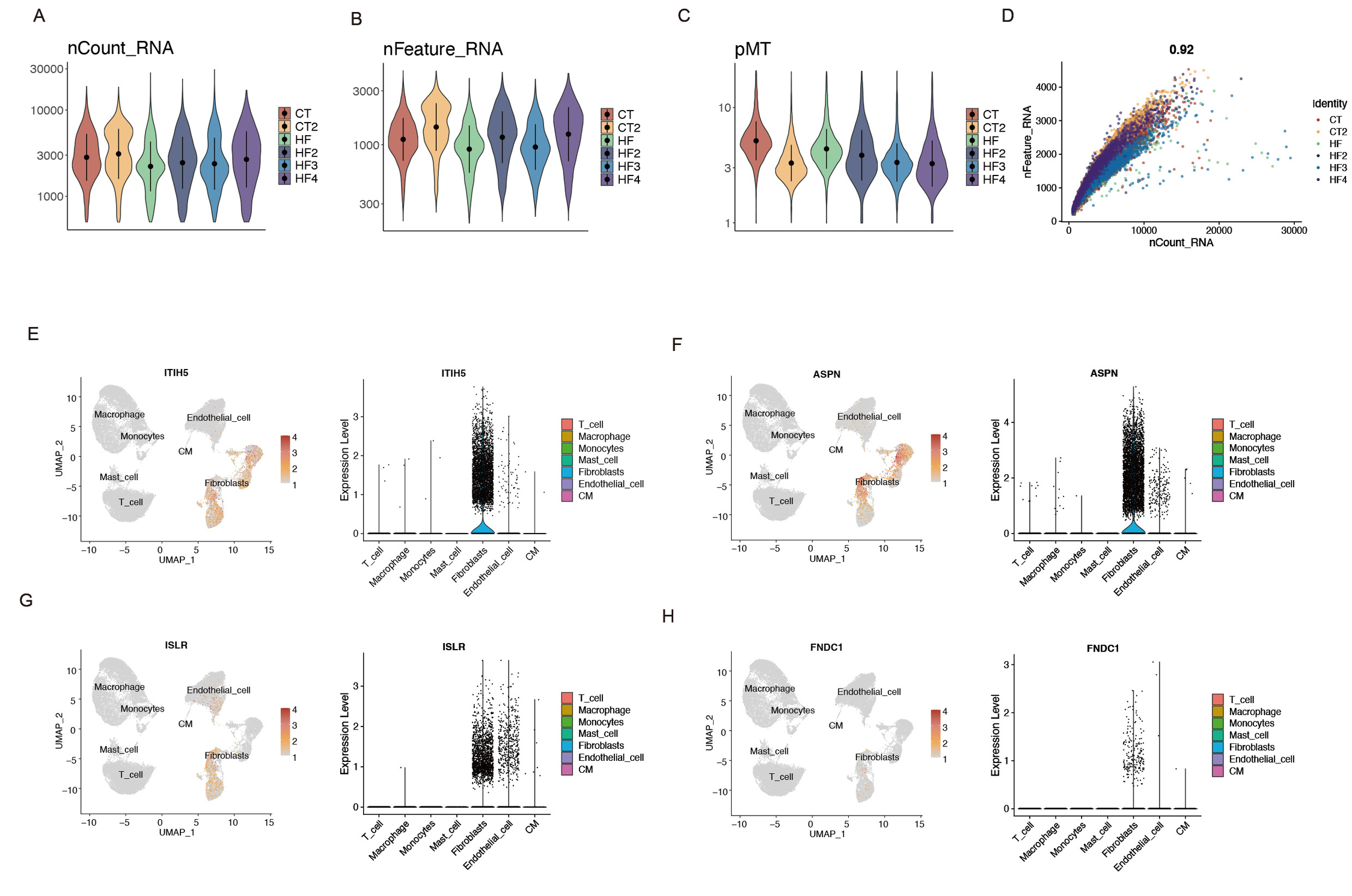

Supplement: S4 Fig — (D) Correlation of gene count of mitochondrial genes and features in HF. (E-H) Distribution of ITIH5, ISLR, ASPN and FNDC1 in each cell type. (TIF) [file pone.0330780.s004.tif]

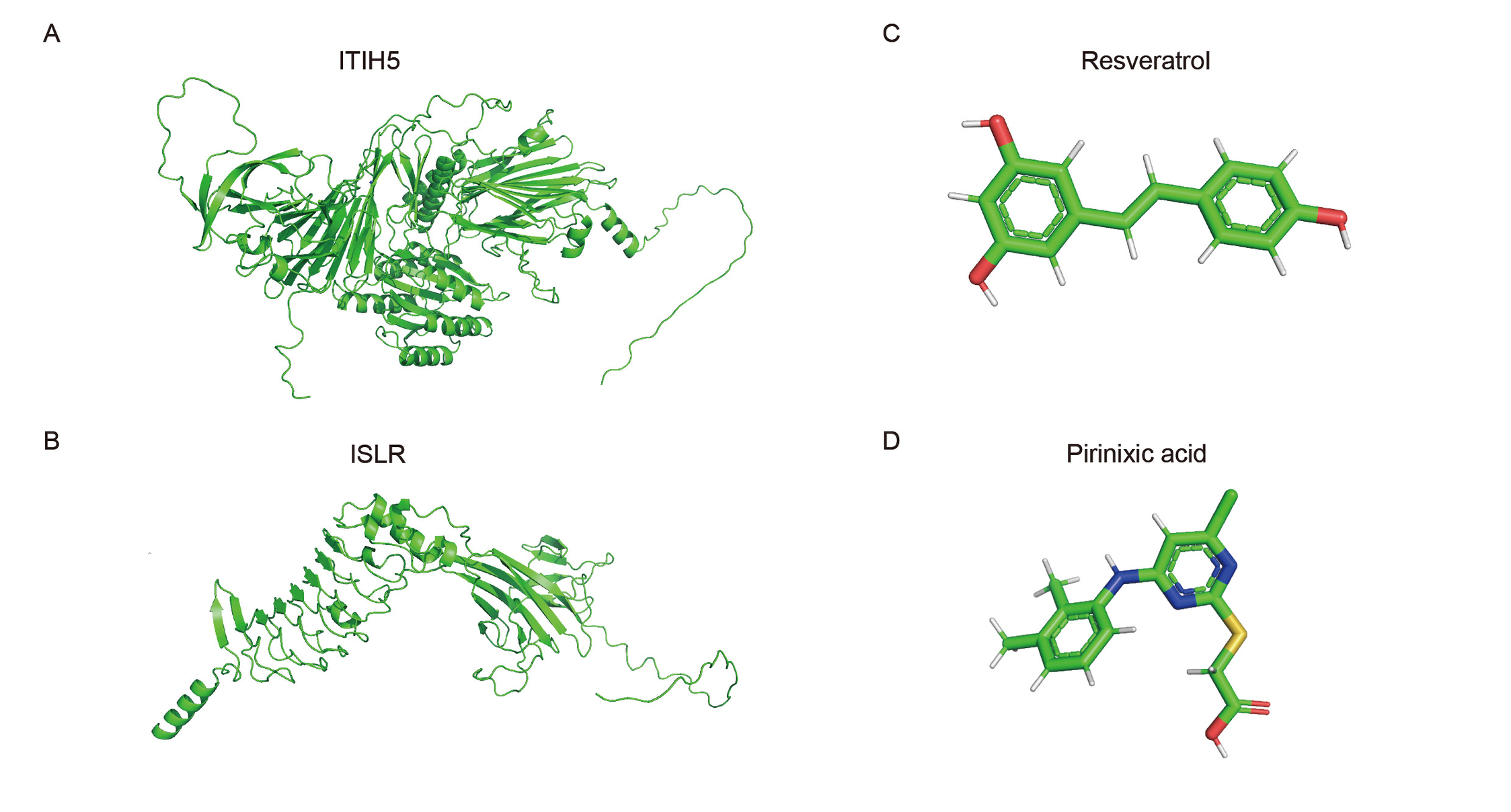

Supplement: S5 Fig — (A) ITIH5 protein structure. (B) ISLR protein structure. (C) Resveratrol chemical Structure. (D) Pirinixic acid chemical structure. (TIF) [file pone.0330780.s005.tif]
