# Supplementary material for: Uncovering key biomarkers, potential therapeutic targets and development of deep learning model in heart failure
Source: PLoS One. 2025 Sep 3;20(9):e0330780. doi: 10.1371/journal.pone.0330780 (PMC12407452; doi:10.1371/journal.pone.0330780)
Supplement: S1 Table — (DOCX) [file pone.0330780.s006.docx]

Supplementary Table 1 The details of the candidate small molecular drugs targeting key genes.

| **Drug name** | **ID** | **Interaction Actions** | **Gene name** |
| --- | --- | --- | --- |
| Bisphenol A | C006780 | Decreases ^ expression | ISLR |
| Carmustine | D002330 | Decreases ^ expression | ISLR |
| Deoxynivalenol | C007262 | Decreases ^ expression | ISLR |
| Dexamethasone | D003907 | Decreases ^ expression | ISLR |
| Doxorubicin | D004317 | Decreases ^ expression | ISLR |
| Ethinyl Estradiol | D004997 | Affects ^ cotreatment \| Decreases ^ expression | ISLR |
| Gentamicins | D005839 | Decreases ^ expression | ISLR |
| Paraquat | D010269 | Decreases ^ expression | ISLR |
| Pirinixic acid | C006253 | Decreases ^ expression | ISLR |
| Schizandrin B | C015499 | Decreases ^ expression \| Decreases ^ reaction | ISLR |
| Sodium arsenite | C017947 | Decreases ^ expression | ISLR |
| T-2 Toxin | D013605 | Decreases ^ expression | ISLR |
| Thioguanine | D013866 | Decreases ^ expression | ISLR |
| Benzo(a)pyrene | D001564 | Decreases ^ expression | ITIH5 |
| Cyclosporine | D016572 | Decreases ^ expression | ITIH5 |
| Deoxynivalenol | C007262 | Decreases ^ expression | ITIH5 |
| Dietary Fats | D004041 | Decreases ^ expression | ITIH5 |
| Doxorubicin | D004317 | Decreases ^ expression | ITIH5 |
| Medroxyprogesterone Acetate | D017258 | Affects ^ cotreatment \| Decreases ^ expression | ITIH5 |
| Ozone | D010126 | Affects ^ cotreatment \| Decreases ^ expression \| Increases ^ abundance | ITIH5 |
| Ozone | D010126 | Decreases ^ expression \| Increases ^ abundance | ITIH5 |
| Paraquat | D010269 | Decreases ^expression | ITIH5 |
| Pirinixic acid | C006253 | Decreases ^ expression | ITIH5 |
| Plant Extracts | D010936 | Affects ^ cotreatment \| Decreases ^ expression | ITIH5 |
| Resveratrol | D000077185 | Affects ^ cotreatment \| Decreases ^ expression | ITIH5 |
| Sodium arsenite | C017947 | Decreases ^ expression | ITIH5 |
| Soot | D053260 | Affects ^ cotreatment \| Decreases ^ expression \| Increases ^ abundance | ITIH5 |
| T-2 Toxin | D013605 | Decreases ^ expression | ITIH5 |
| Tobacco Smoke Pollution | D014028 | Decreases ^ expression | ITIH5 |
| Troglitazone | D000077288 | Decreases ^ expression | ITIH5 |
| Benzo(a)pyrene | D001564 | Affects ^ cotreatment \| Decreases ^ expression | ASPN |
| Benzo(a)pyrene | D001564 | Decreases ^ expression | ASPN |
| Bisphenol A | C006780 | Decreases ^ expression | ASPN |
| Dexamethasone | D003907 | Affects ^ cotreatment \| Decreases ^ expression | ASPN |
| Dibenzothiophene | C016366 | Decreases ^ expression | ASPN |
| Doxorubicin | D004317 | Decreases ^ expression | ASPN |
| Indomethacin | D007213 | Affects ^ cotreatment \| Decreases ^ expression | ASPN |
| Lidocaine | D008012 | Decreases ^ expression | ASPN |
| Particulate Matter | D052638 | Affects ^ cotreatment \| Decreases ^ expression | ASPN |
| Perfluorooctanoic acid | C023036 | Affects ^ cotreatment \| Decreases ^ expression | ASPN |
| Thalidomide | D013792 | Decreases ^ expression | ASPN |
| Valproic Acid | D014635 | Decreases ^ expression | ASPN |
| Arsenic Trioxide | D000077237 | Decreases ^ expression | FNDC1 |
| Benzo(a)pyrene | D001564 | Affects ^ cotreatment \| Decreases ^ expression | FNDC1 |
| Benzo(a)pyrene | D001564 | Decreases ^ expression | FNDC1 |
| Bisphenol A | C006780 | Decreases ^ expression | FNDC1 |
| Cocaine | D003042 | Affects ^ cotreatment \| Decreases ^ expression | FNDC1 |
| Dexamethasone | D003907 | Decreases ^ expression | FNDC1 |
| Doxorubicin | D004317 | Decreases ^ expression | FNDC1 |
| Gentamicins | D005839 | Decreases ^ expression | FNDC1 |
| Ozone | D010126 | Affects ^ cotreatment \| Decreases ^ expression \| Increases ^ abundance | FNDC1 |
| Ozone | D010126 | Decreases ^ expression \| Increases ^ abundance | FNDC1 |
| Paraquat | D010269 | Decreases ^ expression | FNDC1 |
| Particulate Matter | D052638 | Affects ^ cotreatment \| Decreases ^ expression | FNDC1 |
| Soot | D053260 | Affects ^ cotreatment\| Decreases ^expression \| Increases ^ abundance | FNDC1 |
| Soot | D053260 | Decreases ^ expression | FNDC1 |
| T-2 Toxin | D013605 | Decreases ^ expression | FNDC1 |
