# Supplementary material for: Uncovering key biomarkers, potential therapeutic targets and development of deep learning model in heart failure
Source: PLoS One. 2025 Sep 3;20(9):e0330780. doi: 10.1371/journal.pone.0330780 (PMC12407452; doi:10.1371/journal.pone.0330780)
Supplement: S2 Table — (DOCX) [file pone.0330780.s007.docx]

Supplementary Table 2 Molecular docking binding energy of candidate small molecular drugs and targeting key genes.

| **Gene name** | **Drug name** | **Molecular docking binding energy**  **(kcal/mol)** |
| --- | --- | --- |
| ITIH5 | Resveratrol | -6.98 |
| ITIH5 | Pirinixic acid | -6.93 |
| ISLR | Pirinixic acid | -3.28 |
